# Effect of mindfulness-based interventions on people with prehypertension or hypertension: a systematic review and meta-analysis of randomized controlled trials

**DOI:** 10.1186/s12872-024-03746-w

**Published:** 2024-02-14

**Authors:** Qiongshan Chen, Hui Liu, Shizheng Du

**Affiliations:** 1 Shantou University Medical College, 22 Xinling Road, Jinping District, Shantou, Guangdong Province 515041 China; 2 Department of Cardiology, The Second Affiliated Hospital of Shantou University Medical College, 69 Dongxia Road, Jinping District, Shantou, Guangdong Province 515041 China; 3 School of Nursing, Nanjing University of Chinese Medicine, 138 Xianlin Avenue, Qixia District, Nanjing, Jiangsu Province 210023 China

**Keywords:** Anxiety, Blood pressure, Depression, Hypertension, Mindfulness

## Abstract

**Background:**

Hypertension and prehypertension have been widely recognized as the main contributors of global mortality. Evidence shows mindfulness-based interventions may reduce blood pressure and improve mental health. However, the effect of mindfulness-based interventions on blood pressure and mental health has not been fully understood.

**Methods:**

Potential studies published before May 24th 2023 were identified by searching Embase, Ovid Emcare, PsycINFO, CINAHL, Web of Science, Cochrane, PubMed, China National Knowledge Infrastructure, Wanfang database, and VIP China Science. Additionally, two grey databases were searched: Mednar, WorldWideScience.org. The risk of bias in the included studies was assessed using the Cochrane Risk of Bias Assessment tool. The random-effects meta-analyses were conducted using Review Man 5.4 software and the key outcomes are presented as mean difference or standard mean difference and the 95% confidential interval.

**Results:**

Searches returned 802 studies in total, of which 12 were included (*N* = 715). The duration of interventions was 8 weeks in 10 trials and 6 weeks in one trial. Pooled effect sizes indicated reductions in systolic blood pressure (MD = − 9.12, 95% CI [− 12.18, − 6.05], *p* < 0.001), diastolic blood pressure (MD = − 5.66, 95% CI [− 8.88, − 2.43], *p* < 0.001), anxiety (SMD = − 4.10; 95% CI [− 6.49, − 1.71], *p* < 0.001), depression (SMD = − 1.70, 95%CI [− 2.95, − 0.44], p < 0.001) and perceived stress (SMD = − 5.91, 95%CI [− 8.74, − 3.09], p < 0.001) at post-intervention. The findings from subgroup analyses are favorable for mindfulness-based interventions regardless of gender and baseline blood pressure with regard to BP reduction, with a more profound effect observed in participants with higher pre-intervention blood pressure.

**Conclusions:**

The results provide evidence for the positive role of mindfulness-based interventions in hypertension management. More large randomized control trials with sufficient statistical power and long-term follow-up are needed.

**Trial registration:**

The protocol had been registered with Prospero on October 2nd 2021 (registration NO. CRD42021282504).

**Supplementary Information:**

The online version contains supplementary material available at 10.1186/s12872-024-03746-w.

## Background

Hypertension and prehypertension contributes to more than 7.8 million deaths worldwide annually [1] and has been widely recognized as the overriding factor causing cardiovascular diseases (CVDs) and the main contributor of global mortality [2]. Over the past 50 years, the global average blood pressure has experienced zero or mild reduction due to the use of antihypertensive drugs. However, disease control remains poor and there has been a steady increase in the incidence of hypertension, particularly in countries and regions suffering from poverty [3]. According to the analysis of global hypertension data, it is predicted that the number of patients suffering from hypertension will increase to 1.5 billion by 2025 [4]. Under poor control, hypertension could cause or aggravate multiple medical conditions, including coronary heart disease, acute cerebrovascular accident, heart failure and renal insufficiency. As a result, the management of hypertension is a significant economic burden worldwide, especially in low- and middle-income countries, in which the annual loss of approximately 500 billion dollars due to cardiovascular disease including hypertension amounts to approximately 2% of gross domestic product over the period 2011–2025 [5]. To reduce the disease burden of hypertension, concerted efforts are urgently needed to further enhance the awareness, prevention and treatment of this condition [4].

The treatment of hypertension can be generally classified as non-pharmaceutical and pharmaceutical management. Despite the broad efficacy and irreplaceability of antihypertensive drugs, it should be emphasized that medical interventions should be applied simultaneously with non-pharmaceutical interventions [2]. Growing evidence and the latest International Society of Hypertension (ISH) Global Hypertension Practice Guidelines indicate that chronic stress and stressful events are associated with the risk of elevated blood pressure [6–9]. In recognition of this, many guidelines recommend that stress management should be applied as a supplementary intervention for hypertension management [10–12]. Simultaneously, multiple prospective studies and meta-analyses revealed the correlation between anxiety, depression and hypertension [11, 13, 14]. It has been repeatedly proven that unhealthy lifestyle can aggravate psychological disorders and ultimately lead to elevated blood pressure [15]. Fan et al.’s research suggested that lifestyle modifications have positive effects on the management of chronic medical conditions including hypertension [16]. Lifestyle modifications have been recommended as a critical non-pharmacological intervention by four major authorized international institutions [8, 17–19]. Nonetheless, a substantial portion of patients have poor compliance and mere health education is insufficient. Recent studies established the significant role mindfulness-based stress reduction (MBSR) interventions play in lifestyle optimization [20, 21]. ISH guidelines emphasize that mindfulness practice should be assimilated into everyday life [8].

Derived from Buddhist traditions, mindfulness was described as nonjudgmental attention and non-elaborative awareness to experiences in the present moment by Dr. Jon Kabat-Zinn [22]. Mindfulness-based interventions (MBIs) internalize mindfulness in specific forms and aim to foster mindfulness ability. As the earliest form of MBI, MBSR was originally utilized to manage chronic pain in Kabat-Zinn’s outpatient clinics [22], and subsequently developed as a set of standardized group training courses of 8–10 weeks and daily ≥30 minutes of meditation practices [23]. While mindfulness-based cognitive therapy (MBCT) developed by Tess et al. is a kind of psychotherapy that combines cognitive therapy and mindfulness stress reduction therapy and it is used in clinical practice as adjuvant treatment for depression [24]. The core of MBSR and MBCT is mindfulness practice, which involves various methods including body scanning, sitting meditation, mindful walking, mindful yoga, mindful eating, 3-min breathing space, pleasure activities and cognitive restructuring. However, it should be noted that not all mindfulness-related interventions can be understood as MBIs, such as mindfulness meditation and acceptance and commitment therapy (ACT )[25], as there are various differences in philosophy background, main techniques, aims and mechanisms between them and MBIs [26, 27]. Mindfulness is only one component in these practices, while it is the core skill in MBIs, which makes it unreasonable to attribute the health-promoting effects to mindfulness skills specifically. MBSR and MBCT, which shared the same basic program structure, are currently the two widely recognized types of MBIs [28].

MBIs not only contribute to physical and psychological health benefits in the common population [29], but also have been promoted to manage multiple diseases, such as hypertension [30, 31], diabetes [32, 33], anxiety [34–36], insomnia [37–39] and so on. In recent years, growing evidence demonstrated the positive effect of MBIs on hypertension. Multiple researches have shown that MBIs contribute to reductions in blood pressure readings in different study populations, such as breast cancer survivors [40], coronary heart disease [41] and type II diabetics [42]. In addition, MBIs could be employed to alleviate anxiety, depression and psychological stress, which have been found closely linked to elevated blood pressure [13, 43–45]. It may be possibly explained by Mikolasek et al.’s and Rogers et al.’s discoveries that MBIs are effective in developing patients’ capability of self-care, which involves improved compliance to lifestyle interventions and antihypertensive medications, as MBIs can cultivate non-judgmental attitudes or acceptance towards hypertension and raise awareness of all the feasible options [46, 47]. Additionally, two physiological researches on MBIs show a reduction on total peripheral resistance by reducing sympathetic activity and also a reduction on inflammatory markers, thereby lowered blood pressure [48, 49]. MBIs contributed to improve patients’ self-regulation, including diet, physical activity, alcohol, stress reactivity and antihypertensive medication adherence, which are evidence-based determinants of blood pressure [50, 51]. A systematic review including 5 studies showed that the MBSR program has the potential to be a supplementary intervention for lifestyle modification and blood pressure management [31]. This finding is similar with another recent systematic review and meta-analysis including 6 trials from Ciro Conversano et al., in which the researchers found that MBSR was a valid solution to lower blood pressure, especially DBP [30]. However, meta-analyses were not conducted on psychological factors including anxiety, depression and stress. Furthermore, the majority of studies enrolled in above mentioned systematic reviews are relatively small trials with a high degree of heterogeneity in study population and dosage of intervention. Similar clinic BP reduction post-intervention was observed in the meta-analysis of Lee et al., and the effect on DBP was durable within 3 to 6 months after the recruitment [52]. However, the diagnosis of prehypertension or hypertension was not mandatory in some of the included studies. The inclusion of normotensive participants might cause bias because of different response to MBIs between hypertensive or prehypertensive patients and normotensive participants. Thus, the treatment effect of MBIs on hypertension or prehypertension is yet to be fully understood.

Therefore, the current systematic review and meta-analysis is necessary to further verify the impact of MBIs as supplementary interventions for patients with hypertension or prehypertension. It will provide more evidence for hypertension management.

## Methods

### Design

The protocol for this systematic review and meta-analysis was designed after consulting with an expert in MBCT and MBSR and an expert in evidence-based nursing. The protocol had been registered with Prospero on October 2nd 2021 (registration NO. CRD42021282504).

### Search strategy

Ten electronic databases were systematically searched: Embase, Ovid Emcare, PsycINFO, CINAHL, Web of Science, Cochrane and PubMed, China National Knowledge Infrastructure (CNKI), Wanfang databases, and VIP China Science. Additionally, two grey databases were searched: Mednar, WorldWideScience.org. References of the collected articles were also screened in search of additional qualified studies. The search strategy was combined Medical Subject Headings (MeSH) and text words. The following search terms were included: (“Hypertension (MeSH)” OR “High Blood Pressure” OR “prehypertension (MeSH)” OR “elevated blood pressure”) AND (“mindfulness (MeSH)” OR “mindfulness-based intervention*” OR “mindfulness-based stress reduction” OR “MBSR” OR “mindfulness-based cognitive therapy” OR “MBCT”) AND (“randomized controlled trial” OR “randomized” OR “RCT” OR “controlled clinical trial” OR “random allocation” OR “randomly”). Studies published between 1979 and 24th May 2023 were searched (see Supplementary Material Table S1). Grey literature searches were conducted on Mednar and worldwidescience.org , and the search strategies were designed by combining key synonyms for “mindfulness”, “hypertension”, “prehypertension” and “randomized controlled trial” with the Boolean operators “AND” and “OR”.

### Study selection

The included studies were selected in accordance with standard approaches recommended by PRISMA flowcharts and Endnote X9 was used to screen studies. Two independent reviewers screened articles respectively against the eligibility criteria and checked the included articles together. All articles were evaluated respectively by two independent reviewers in two steps. At the first step, duplicates were removed by Endnote automatically or manually. Subsequently, two reviewers selected articles per the inclusion criteria and exclusion criteria by titles and abstracts. At the second step, full texts were searched and assessed. The results from two reviewers were checked and compared at each step. The controversial articles were assessed by the third reviewer to reach an agreement.

### Eligibility criteria

The inclusion criteria in this study were:

1. Participants: Age ≥ 18 years old; patients with prehypertension or hypertension (SBP ≥ 120 mmHg and/or DBP ≥ 80 mmHg) (corresponds to the guidelines of the American Heart Association )[53, 54]; with or without prescribed antihypertensive medications;
2. Intervention: Interventional studies on MBIs, including MBSR, MBCT or structured training programs on mindfulness to teach a series of mindfulness practices, e.g., body scanning, mindful walking, and sitting meditations.
3. Comparator: The intervention group is compared with a wait-list or control group including active control group (a group of participants who received an intervention, different from mindfulness, at the same time) or treatment as usual.
4. Outcome measures: Both physical outcomes and psychological outcomes from randomized controlled trials were considered. Physical outcomes included SBP and DBP, which were the primary outcomes. Blood pressure could be measured by different methods, including clinic BP (measured in sitting position in the office or clinic) and ambulatory BP. And blood pressure could be measured by different devices, including automated oscillometric BP devices and manual sphygmomanometer. Psychological outcomes included anxiety, depression and perceived stress, which were the secondary outcomes. And the secondary outcomes could be measured using standardized rating scales [such as the Hospital Anxiety and Depression Scale (HADS), the Depression, Anxiety and Stress Scales (DASS-21), the Perceived Stress Scale (PSS), etc].
5. Study design: Randomized controlled trials (RCTs).
6. Language: Studies published in English or Chinese language.
7. Publication timeframe: Studies published from 1979 year to May 24th 2023.
8. Type of article: Full-text available.

The exclusion criteria in this study were:

1. The participants were pregnant or lactating women; or with previous experience of mindfulness, meditation or similar techniques.
2. Studies which have more comprehensive data were selected if the studies were published repeatedly.

### Data collection process

An independent reviewer (Q.C.) collected data from all articles by using a standardized and piloted Excel table. Another reviewer (H.L.) verified and confirmed data the first reviewer entered into the Excel table. Any divergence was discussed with the third independent reviewer (S.D.) until a consensus of opinion was reached. For the literature lacking data, the author tried to reach the original author for supplementary information. The following data was extracted: 1) Characteristics of the included articles: first authors’ surname, publication year, country and study designs. 2) Characteristic of the participants: study population, the sample size, percentage of intervention group and control group, percentage of females, average age and standard deviation. 3) Details of interventions and control group; 4) Outcome data: primary outcomes and secondary outcomes, assessment time points.

### Risk of bias assessment in included studies

Risk of bias was evaluated by employing the Risk of Bias tool (version 1) for RCT recommended in Cochrane Collaboration 5.1 Version [55]. The assessment focused on a set of domains of bias: (1) Randomization and randomized sequence generation; (2) Allocation concealment; (3) Blinding for participants and outcome assessors; (4) Outcome assessment and measurement; (5) Selected report; (6) Other bias. The risk of bias assessment was completed by two reviewers (S.D. and Q.C.) independently and any discrepancy was eliminated through discussion with the third reviewer (H.L.). Each domain can be judged as‘Low’, or‘High’ risk of bias, or ‘Unclear’. It was judged to be at low risk of bias if all domains were estimated as low risk of bias, at high risk of bias if at least one domain was regarded as high risk of bias, and as unclear if at least one domain was assessed as unclear except that none of domains was judged to be at high risk of bias.

### Data synthesis and analysis

The Review Man 5.4 software developed by Cochrane network was utilized for meta-analysis. The outcome variables were continuous variables, so mean difference (MD) and standard mean difference (SMD) were used as the effect index, and the point estimates and 95% CI of each effect were given [56]. MD is a standard statistic that measures the absolute difference between the mean value in two groups of a randomized trial (e.g. blood pressure). SMD is used as a summary statistic when the studies all assess the same outcome, but measure it in a variety of ways (e.g. depression measured using different psychometric scales )[57]. Of note, as different scales were adopted to measure psychological outcomes, we used SMD for the corresponding meta-analyses. The data of median, maximum and minimum mentioned in the included study were transformed according to the formula and then combined for analysis [58]. The heterogeneity included in the study was analyzed by chi-square test (the test level was a = 0.1), and combined with I^2^ statistics for evaluation. Heterogeneity examination was used to assess statistical heterogeneity between studies [59]. Considering a possible heterogeneity in the included studies, random-effects model was used for meta-analysis. Subgroup analyses were conducted to investigate the differential effects of MBIs on different participants and to explore heterogeneity. These factors included sample size, the type of control group, baseline BP, gender, use of antihypertensive drugs, the type of MBIs, the source of population. *P* < 0.05 was regarded as statistically significant. Sensitivity analysis was to conduct meta-analyses using “leave-one-out” method, and compare the difference between the results after exclusion and the initial combined results. Reporting bias was intuitively judged by drawing funnel diagram and Stata 14.0 software for quantitative evaluation by Egger’s test.

## Results

### Search results

**Fig. 1:**
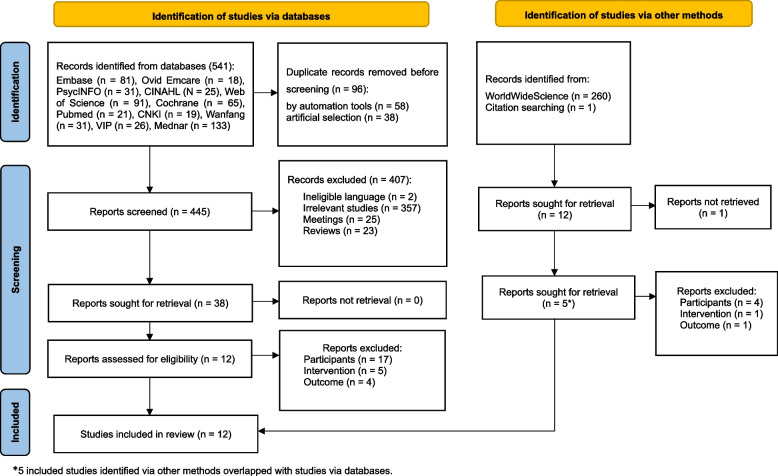
PRISMA flowchart for literature search and selection

Eight hundred and two articles in total were preliminarily searched and one additional article was retrieved from citations of the included studies. Ninety-six duplicates were removed and 407 articles were screened according to title, abstract, type of publication and language with 38 articles left. Eventually, 12 articles were enrolled in the systematic review. Five included studies identified via the WorldwildeScience.org and citation searching overlapped with studies from databases. A PRISMA flowchart of search results and study selections is depicted in Fig. 1.

### Risk of bias assessment

**Fig. 2:**
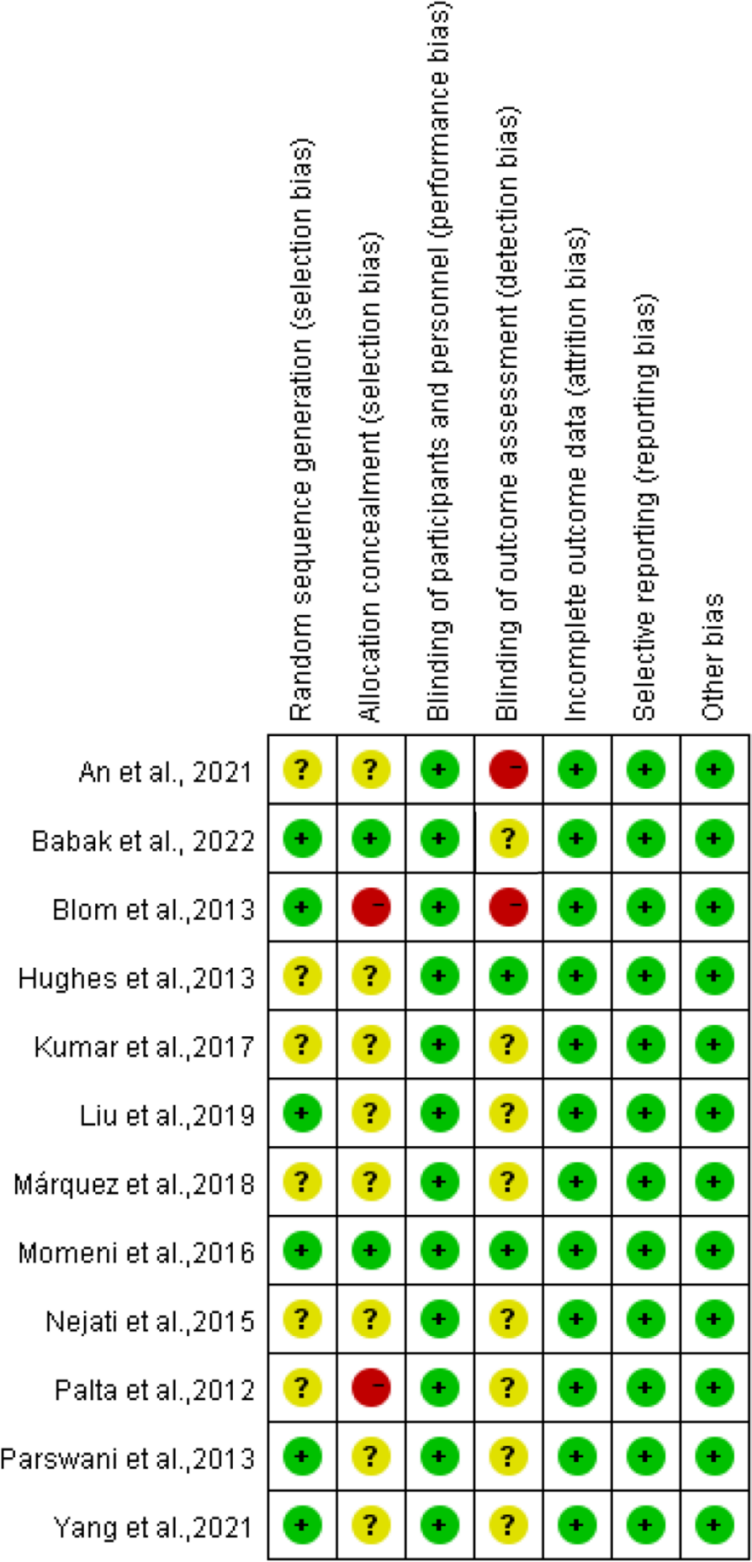
Risk of bias of the selected literature assessed by RoB(version1)

The result of risk of bias assessment is shown in Fig. 2. All articles mentioned randomization but only six reported how the random sequence was generated [41, 60–64]. Most of the studies had an unclear risk of bias for allocation concealment, as only one study gave a description of concealment procedures [62]. Given the nature of psychological intervention experiments, blinding of participants was not possible to achieve. Most of the participating patients were aware of the study group assignment. For most of the studies, the information of the blinding of outcome assessors was scarcely provided, as only two studies clearly stated that assessors were blinded to treatment allocation [62, 65]. All the study outcomes were reported in the prespecified way. With regard to funding, 6 RCTs were funded but the authors stated no interest conflict.

### Study characteristics

A total of twelve RCTs were conducted in America (*n* = 3) [21, 65, 66], Canada (*n* = 1) [63], India (*n* = 2) [41, 67], Iran (*n* = 3) [20, 62, 64], Spain (*n* = 1) [68] and China (*n* = 2) [60, 61] respectively. Ten articles were written in English and two were in Chinese. The sample size varied from 20 to 113 and 715 participants in total were allocated. Participants received 8 weekly sessions of structured MBSR intervention in 10 studies, MBCT which consisted of weekly sessions over 8 weeks in one study [68] and 6 weeks of Mindful Awareness Practice (MAP) in one research [21]. While intervention in the control group was treatment as usual in four trials [41, 60, 61, 64], wait-list in three trials [62, 63, 67] and active treatment in six trials including social support [66], progressive muscle relaxation (PMR) training [65], yoga training [20], health education [21, 68] (see Supplementary Material Table S2).

### Study populations

Patients were recruited from hospitals and private clinics in 6 studies [20, 41, 61, 62, 64, 68]. Six studies recruited participants from communities [21, 60, 63, 65–67]. All but two trials (100% male in Parswani et al.’s study [41] and 100% female in Babak et al.’s study [64]) were mixed-gender, with females accounting for 40 to 95%. The average age of participants ranged from 43.13 to 73.7 years old. Two out of the 12 studies included unmedicated patients diagnosed with prehypertension or grade-1 hypertension [63, 65]. Nine trials enrolled prehypertensive or hypertensive participants, most of whom were medicated, among which three studies recruited hypertension (see Supplementary Material Table S2).

### Intervention characteristics

Of the 12 studies, ten adopted Kabat-Zinn’s theoretical framework of 8 weekly sessions of MBSR in the design of their intervention. One study [68] applied 8 weekly sessions of MBCT, in accordance with the approach of Segal et al. Another one used 6 weekly sessions of MAP based on mindfulness skills [21]. All the activities were done in a group setting and lasted 45 minutes to 2.5 hours each time. Five to 45 minutes of home practice was required in 7 studies [21, 41, 60, 62, 63, 65, 66, 68]. Except for 4 studies, other researchers reported the qualification of instructors of MBSR, including therapists trained for MBSR [63–66], a licensed psychologist [62], an psychiatrist trained for MBCT [68], certified instructors [21] and nurses [61]. The follow-up time ranged from immediately post-intervention to 3 months after intervention (see Supplementary Material Table S3).

### Meta-analysis outcomes

#### Physical outcome - blood pressure

**Fig. 3:**
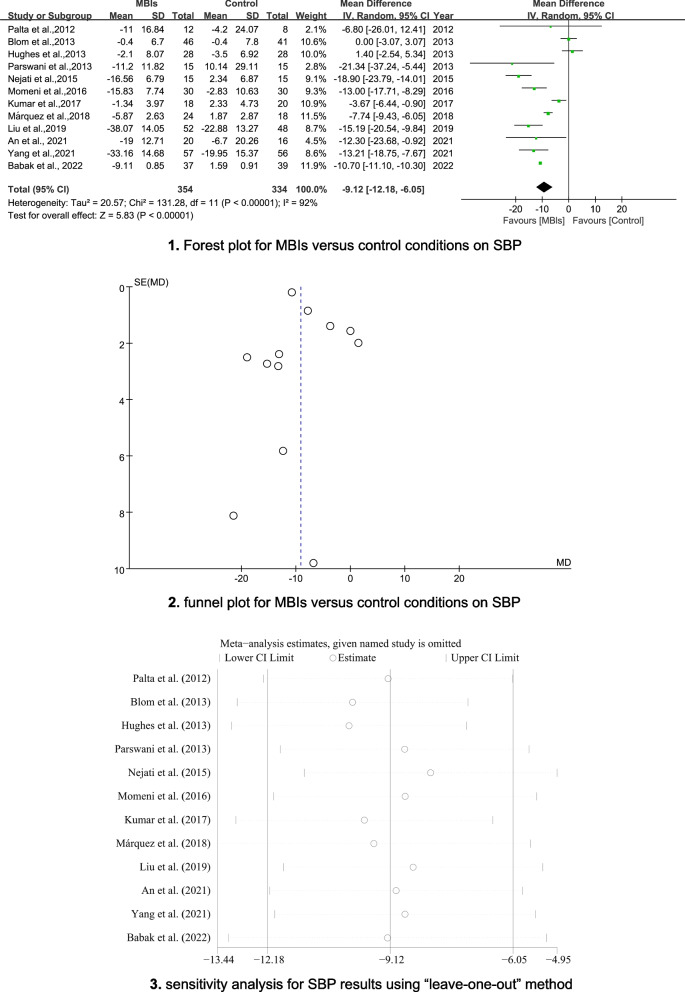
3-1 Forest plot for MBIs versus control conditions on SBP. 3-2 Funnel plot for MBIs versus control conditions on SBP. 3-3 Sensitivity analysis for SBP results using “leave-one-out” method

**Fig. 4:**
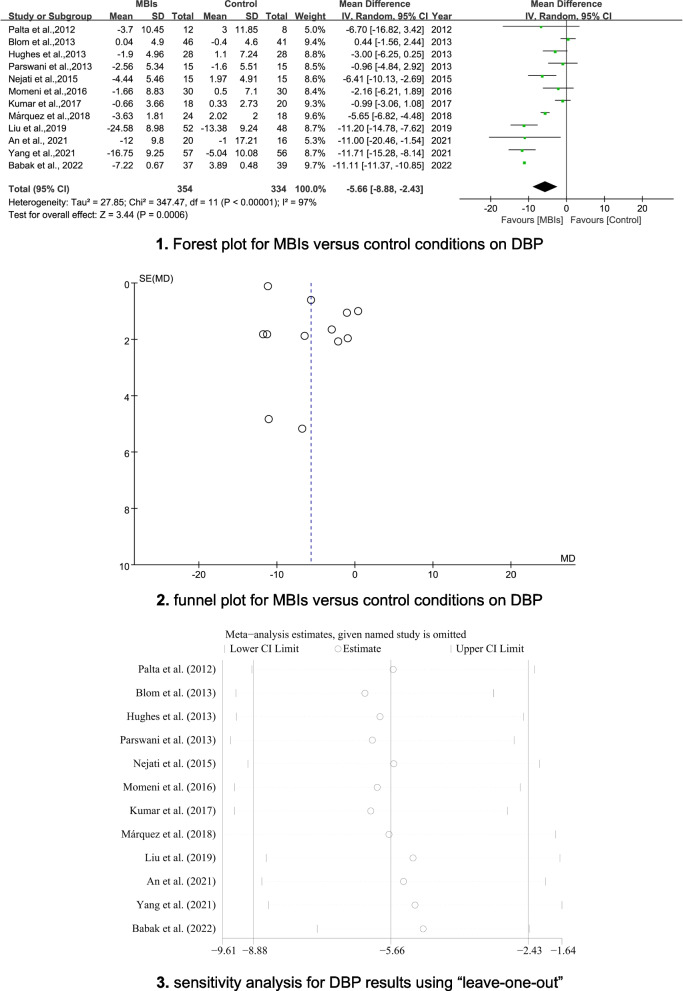
4-1 Forest plot for MBIs versus control conditions on DBP. 4-2 Funnel plot for MBIs versus control conditions on DBP. 4-3 Sensitivity analysis for DBP results using “leave-one-out” method

The pooled results indicated statistically significant effects of MBIs for reducing SBP and DBP (MD = -9.12; 95% CI [− 12.18, − 6.05], *p* < 0.001, I^2^ = 92%; MD = − 5.66; 95% CI [− 8.88, − 2.43], p < 0.001, I^2^ = 97%, respectively). The forest plots are depicted in Figs. 3-1 and 4-1. Egger’s tests indicated the absence of publication bias (*p* = 0.235; *p* = 0.696). The funnel plots are shown in Figs. 3-2 and 4-2. Sensitivity analyses demonstrated that the results of SBP or DBP were not impacted (see Figs. 3-3 and 4-3).

Of the 12 studies, 6 had a sample size of 50 or more, so we included these studies in the subgroup analyses for MBIs on SBP and DBP, respectively. The results indicated a high heterogeneity and statistically significant mean effect sizes favoring MBIs (SBP: MD = − 8.25, 95% CI [− 13.47, − 3.03], *p* = 0.002, I^2^ = 94%, Supplementary Material Fig. A-1; DBP: MD = − 6.46; 95% CI [− 11.57, − 1.36], *p* = 0.01, I^2^ = 97%, Supplementary Material Fig. B-1). Meanwhile, meta-analyses including trials with a sample size of less than 50 showed a decreased heterogeneity and statistically significant mean effect sizes favoring MBIs (SBP: MD = − 10.71; 95% CI [− 15.91, − 5.51], *p* < 0.001, I^2^ = 84%, Supplementary Material Fig. A-1; DBP: MD = − 4.22; 95% CI [− 6.98, − 1.47], *p* = 0.003, I^2^ = 76%, Supplementary Material Fig. B-1). The comparisons of MBIs against different controls (wait-list, treatment-as-usual, and active control) obtained varying results. When comparing MBIs to wait-list control, the effect sizes on BP were not statistically significant (SBP: *p* = 0.10, I^2^ = 90%, Supplementary Material Fig. A-2; DBP: *p* = 0.50, I^2^ = 0%, Supplementary Material Fig. B-2). When comparing MBIs to active control conditions such as social support, PMR training, yoga training, and health education, statistically significant mean effect sizes on BP were observed (SBP: MD = − 8.78; 95% CI [− 15.98, − 1.57], *p* = 0.02, I^2^ = 90%, Supplementary Material Fig. A-2; DBP: MD = − 8.94; 95% CI [− 12.89, − 5.00], *p* < 0.001, I^2^ = 89%, Supplementary Material Fig. B-2). The comparisons of MBIs to treatment-as-usual showed significant mean effect sizes on BP (SBP: MD = − 12.44; 95% CI [− 15.41, − 9.46], p < 0.001, Supplementary Material Fig. A-2; DBP: MD = − 5.51; 95% CI [− 6.56, − 4.47], p < 0.001, Supplementary Material Fig. B-2), with a lower heterogeneity (SBP: I^2^ = 42%; DBP: I^2^ = 0%).

The subgroup analysis based on baseline BP demonstrated statistically significant BP reductions in both subgroups. However, the studies that enrolled participants with higher baseline SBP (≥140 mmHg)/ DBP (≥90 mmHg) showed greater reductions (SBP: MD = − 11.68; 95% CI [− 15.81, − 7.55], *p* < 0.001; MD = − 6.79; 95% CI [− 12.01, − 1.57], *p* = 0.01; Supplementary Material Fig. A-3. DBP: MD = − 7.47; 95% CI [− 13.19, − 1.75], p = 0.01; MD = − 4.68; 95% CI [− 8.74, − 0.61], *p* = 0.02; Supplementary Material Fig. B-3). Subgroup analyses based on female proportion revealed that both subgroups had statistically significant mean effect sizes on BP. However, the studies that included more males demonstrated greater SBP reductions (MD = − 15.30; 95% CI [− 17.85, − 12.74], *p* < 0.001) and a much lower heterogeneity (I^2^ = 3%) than those with more females (MD = − 5.08; 95% CI [− 9.13, − 1.04], p = 0.01; I^2^ = 95%) (Supplementary Material Fig. A-4). Similar effect sizes were observed with regard to DBP (males: MD = − 5.02; 95% CI [− 9.49, − 0.54], *p* = 0.03, I^2^ = 98%; females: MD = − 6.55; 95% CI [− 10.90, − 2.20], *p* = 0.003, I^2^ = 85%; Supplementary Material Fig. B-4).

Subgroup analysis based on antihypertensive medication status demonstrated mixed results, with a statistically significant mean effect size on SBP/DBP favoring MBIs observed in the studies that recruited medicated patients (SBP: MD = − 15.30; 95% CI [− 17.85, − 12.74], *p* < 0.001, I^2^ = 3%, Supplementary Material Fig. A-5; DBP: MD = − 6.55; 95% CI [− 10.90, − 2.20], p = 0.003, I^2^ = 85%, Supplementary Material Fig. B-5). While studies that included both medicated and unmedicated participants showed a smaller effect size of MBIs on SBP/DBP (SBP: MD = − 7.90; 95% CI [− 11.22, − 4.95], *p* < 0.001, I^2^ = 88%, Supplementary Material Fig. A-5; DBP: MD = − 6.71; 95% CI [− 11.44, − 1.99], *p* = 0.005, I^2^ = 98%, Supplementary Material Fig. B-5) and studies that included unmedicated patients showed no lowering effect of MBIs on SBP/DBP (SBP: MD = 0.53; 95% CI [− 1.89, 2.95], *p* = 0.67, I^2^ = 0%, Supplementary Material Fig. A-5; DBP: MD = − 1.03; 95% CI [− 4.37, 2.30], *p* = 0.54, I^2^ = 68%, Supplementary Material Fig. B-5). The subgroup analysis based on the type of MBIs indicated statistically significant BP reductions in MBSR with a high heterogeneity (SBP: MD = − 9.36; 95% CI [− 13.44, − 5.29], *p* < 0.001, I^2^ = 93%, Supplementary Material Fig. A-6; DBP: MD = -5.33; 95% CI [− 9.40, − 1.26], *p* = 0.01, I^2^ = 97%, Supplementary Material Fig. B-6). Subgroup analysis based on the source of patients demonstrated larger reductions on BP in patients recruited from hospitals and private clinics instead of communities (SBP: MD = − 12.04; 95% CI [− 14.66, − 9.43], p < 0.001, I^2^ = 81%, Supplementary Material Fig. A-7; DBP: MD = − 5.07; 95% CI [− 10.10, − 0.04], *p* < 0.001, I^2^ = 84%, Supplementary Material Fig. B-7).

### Psychological outcome - anxiety, depression and perceived stress

**Fig. 5:**
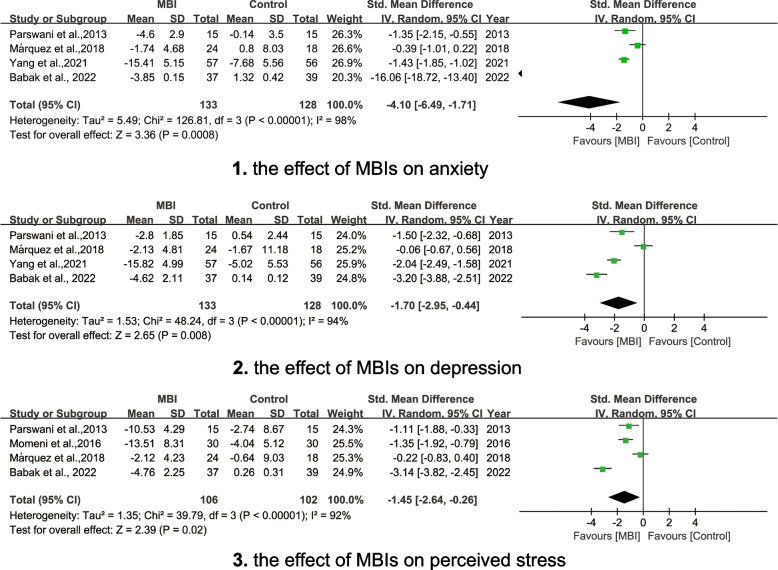
5-1 The effect of MBIs on anxiety. 5-2 The effect of MBIs on depression. 5-3 The effect of MBIs on perceived stress

Overall, MBIs participation resulted in statistically significant favorable effects for psychological outcomes across populations diagnosed with hypertension. Using pooled data, statistically significant and beneficial effects on anxiety (SMD = − 4.10; 95% CI [− 6.49, − 1.71], *p* = 0.002) (see Fig. 5-1) and depression (SMD = − 1.70, 95%CI [− 2.95, − 0.44], *p* = 0.008) (see Fig. 5-2) were observed across four studies (*n* = 261). The effect across four studies (*n* = 208) was observed with a statistically significant effect for perceived stress (MD = − 1.45, 95%CI [− 2.64, − 0.26], *p* = 0.02) (see Fig. 5-3).

## Discussion

The present meta-analysis included a total of 12 RCTs and provided evidence that a structured MBI program can help reduce SBP and DBP in prehypertensive or hypertensive individuals. However, considerable heterogeneity was found among the studies included, indicating the need for caution when drawing conclusions from these findings.

Comparing with Conversano et al.’s review [30], we searched more databases and included 6 more studies and involved a total of 715 participants. Our study further confirms the result from Conversano et al.’s research that MBI is a promising strategy for hypertension both in SBP and DBP. However, our findings provide evidence of a greater reduction in SBP. This is consistent with Lee et al.’s review on the effect of MBSR on blood pressure [52]. In addition, the results also support previous studies which showed that MBIs can lower blood pressure in patients with other different diseases, such as breast cancer survivors [40], coronary heart disease [41] and diabetic patients [42]. The findings from subgroup analyses are favorable for MBIs regardless of gender and baseline blood pressure, with a more profound effect observed in males and participants with higher pre-intervention blood pressure.

According to control intensity, wait-list control means blank control, treatment as usual is a standard control, and positive intervention such as yoga and exercise is equivalent to active control [69]. Usually, the adoption of a wait-list control would result in differences in expectancy effects and therefore overestimate the treatment effect. In the present meta-analysis, however, subgroup analysis based on the type of control group demonstrated that the overall antihypertensive effect of MBIs was not statistically significant in trials employing a wait-list control, while the use of an active control or treatment-as-usual led to positive treatment effects on SBP and DBP reductions. It seems that our findings did not support that active control interventions such as relaxation and exercise (which are also related to stress reduction) may undermine relative benefits from MBIs. This can be partly explained by that the largest one among the three studies using a wait-list control published by Blom et al. [63] enrolled unmedicated hypertensive patients and obtained negative result. Subgroup analyses from the current meta-analysis also indicated that studies focusing on untreated hypertension resulted in a non-significant BP lowering effect of MBIs compared with control conditions. As MBIs may have an indirect impact on BP by improving drug compliance, unmedicated patients might benefit less from these therapies. Nonetheless, it should be noted that only two studies included unmedicated participants, which might underestimate the positive effect MBIs have on blood pressure. In addition, an interesting finding is that the effect of MBIs on blood pressure was more profound in males, which is inconsistent with previous meta-analyses. According to a recent research, males tend to have lower awareness and poorer control of hypertension [70]. A possible explanation is that MBIs can help lower blood pressure by raising their awareness and understanding of the disease and thus provide more benefits for them.

Due to the lack of standardized procedure and settings of MBIs employed in the included RCTs, considerable variation was observed in the specific methods and duration of interventions administered. In addition, most studies identified were small trials of varying geographical population with differences in race and stage of hypertension.

In terms of psychological outcomes, the results are similar with a previous systematic review on the impact of MBSR and MBCT on the psychosocial well-being of patients with vascular conditions [71]. Our findings suggest that mindfulness therapy exerts a positive impact on anxiety, depression and psychosocial stress. However, the sample size was relatively small, as there were only four articles that reported anxiety, perceived stress and depression in our review. More studies are warranted to provide further evidence.

### Limitations

Most studies included in the current research were of moderate to high level of risks and only one study was identified as low level of risks, which undermines the evidence provided. In addition, some studies included had small sample sizes which likely reduced statistical power. The safety profile of mindfulness-based interventions was not analyzed due to the possible under-reporting of adverse events caused by MBIs in these researches, as only one study reported a serious adverse event which occurred in the control group [63]. Only one study recruited patients with grade II hypertension (BP ≥ 160/100 mmHg), which sets limitation on the generalizability of the results to this patient population. Besides, only articles published in English and Chinese were identified as these are the only two readable languages to the reviewers.

## Conclusions and future perspectives

Our meta-analysis suggests that MBIs can reduce blood pressure, anxiety, depression and perceived stress, which supports the use of MBIs as supplementary interventions for prehypertensive and hypertensive populations, preferably in combination with antihypertensive medications. Despite these encouraging findings, more randomized controlled trials with larger sample size and better study design are warranted to provide higher-quality evidence.

### Supplementary Information

**Additional file 1.** Supplementary Material.

## Data Availability

All data generated or analyzed in this study are openly available on Mendeley Data at 10.17632/bf7crz6mvk.1.

## Abbreviations

CVDs: Cardiovascular diseases
ISH: International Society of Hypertension
MBSR: Mindfulness-based stress reduction
MBIs: Mindfulness-based interventions
MBCT: Mindfulness-based cognitive therapy
ACT: Acceptance and commitment therapy
SBP: Systolic blood pressure
DBP: Diastolic blood pressure
PRISMA: Preferred reporting items for systematic reviews and Meta-analyses
HADS: Hospital anxiety and depression scale
DASS: Depression, anxiety and stress scales
PSS: Perceived stress scale
RCTs: Randomized controlled trials
MD: Mean difference
SMD: Standard mean difference
CI: Confidence interval
MAP: Mindful awareness practice
PMR: Progressive muscle relaxation
SAS: Self-rating anxiety scale
SDS: Self-rating depression scale
