# Supplementary material for: Effect of mindfulness-based interventions on people with prehypertension or hypertension: a systematic review and meta-analysis of randomized controlled trials
Source: BMC Cardiovasc Disord. 2024 Feb 14;24:104. doi: 10.1186/s12872-024-03746-w (PMC10865530; doi:10.1186/s12872-024-03746-w)

**Supplementary Material**

**Table S1. Search strategy**

Search strategy for Embase：

| #1 | ‘ Hypertension’ /exp |
| --- | --- |
| #2 | ‘Blood Pressure, High’ :ab,ti OR ‘Blood Pressures, High’ :ab,ti OR ‘High Blood Pressure’ :ab,ti OR ‘High Blood Pressures’ :ab,ti OR ‘elevated blood pressure’ :ab,ti |
| #3 | ‘Prehypertension’ /exp |
| #4 | ‘Prehypertension’ :ab,ti |
| #5 | ‘Mindfulness’ /exp |
| #6 | mindfulness-based intervention* :ab,ti OR mindfulness-based stress reduction :ab,ti OR MBSR :ab,ti OR mindfulness-based cognitive therapy :ab,ti OR MBCT :ab,ti |
| #7 | ‘randomized controlled trial’ :it OR randomi?ed :ab,ti OR RCT :ab,ti  ‘controlled clinical trial’ :ab,ti OR ‘random allocation’ :ab,ti OR randomly :ab,ti |
| #8 | #1 OR #2 OR #3 OR #4 |
| #9 | #5 OR #6 |
| #10 | #7 AND #8 AND #9 |

Search strategy for Web of Science:

| #1 | ((((((((TS= (hypertension)) OR TS= (Blood Pressure, High)) OR TS= (Blood Pressures, High)) OR TS= (High Blood Pressure)) OR TS= (High Blood Pressures)) OR TS= (elevated blood pressure)) OR TS= (Prehypertension)) OR TS= (Pre-Hypertension)) OR TS= (Pre Hypertension) |
| --- | --- |
| #2 | (((((TS= (Mindfulness)) OR TS= (mindfulness-based intervention*)) OR TS= (mindfulness-based stress reduction)) OR TS= (MBSR)) OR TS= (mindfulness-based cognitive therapy)) OR TS= (MBCT) |
| #3 | (((((TS= (Randomized controlled trial)) OR TS= (randomi?ed)) OR TS=(RCT)) OR TS= (controlled clinical trial)) OR TS= (random allocation)) OR TS= (randomly) |
| #4 | #1 OR #2 OR #3 |

Search strategy for Pubmed：

| #1 | Hypertension [MeSH] |
| --- | --- |
| #2 | (Blood Pressure, High [Title/Abstract] OR Blood Pressures, High [Title/Abstract] OR High Blood Pressure [Title/Abstract] OR High Blood Pressures [Title/Abstract] OR elevated blood pressure [Title/Abstract]) |
| #3 | Prehypertension [MeSH] |
| #4 | (Pre-Hypertension [Title/Abstract] OR Pre Hypertension [Title/Abstract]) |
| #5 | #1 OR #2 OR #3 OR #4 |
| #6 | Mindfulness [MeSH] |
| #7 | (mindfulness-based intervention* [Title/Abstract] OR mindfulness-based stress reduction [Title/Abstract] OR MBSR [Title/Abstract] OR mindfulness-based cognitive therapy [Title/Abstract] OR MBCT [Title/Abstract]) |
| #8 | #6 OR #7 |
| #9 | Randomized controlled trial [Publication Type] OR randomized [Title/Abstract] OR RCT [Title/Abstract] OR controlled clinical trial [Title/Abstract] OR random allocation [Title/Abstract] OR randomly [Title/Abstract] |
| #10 | #5 AND #8 AND #9 |

Search strategy for Cochrane:

| #1 | MeSH descriptor: [Hypertension] explode all trees |
| --- | --- |
| #2 | (Blood Pressure, High) :ti ,ab,kw OR (Blood Pressures, High) :ti ,ab,kw OR (High Blood Pressure) :ti ,ab,kw OR (High Blood Pressures) :ti ,ab,kw OR (elevated blood pressure) :ti ,ab,kw |
| #3 | MeSH descriptor: [Prehypertension] explode all trees |
| #4 | MeSH descriptor: [Mindfulness] explode all trees |
| #5 | (mindfulness-based intervention*):ti ,ab,kw OR (mindfulness-based stress reduction):ti ,ab,kw OR (MBSR):ti ,ab,kw OR (mindfulness-based cognitive therapy):ti ,ab,kw OR (MBCT):ti ,ab,kw |
| #6 | (randomized controlled trial):ti ,ab,kw OR (randomi?ed):ti ,ab,kw OR (RCT):ti ,ab,kw (controlled clinical trial):ti ,ab,kw OR (random allocation):ti ,ab,kw |
| #7 | (randomly):ti ,ab,kw |
| #8 | #1 OR #2 OR #3 |
| #9 | #4 OR #5 |
| #10 | #6 OR #7 |
| #11 | #8 AND #9 AND #10 |

Search strategy for CINAHL：

| S1 | MH Hypertension OR Blood Pressure, High OR Blood Pressures, High OR MH High Blood Pressure OR High Blood Pressures OR elevated blood pressure |
| --- | --- |
| S2 | MH Prehypertension MH Pre-Hypertension OR Pre Hypertension |
| S3 | MH Mindfulness MH mindfulness-based intervention* OR MH mindfulness-based stress reduction OR MH MBSR OR MH mindfulness-based cognitive therapy OR MH MBCT |
| S4 | PT Randomized controlled trial OR randomized OR RCT OR MH controlled clinical trial OR random allocation OR randomly |
| S5 | S1 OR S2 |
| S6 | S3 OR S4 OR S5 |

Search strategy for Ovid Emcare：

| S1 | (Hypertension OR Blood Pressure, High OR Blood Pressures, High OR High Blood Pressure OR High Blood Pressures OR elevated blood pressure).mp.[mp=title, abstract, heading word, drug trade name, original title, device manufacturer, device trade name, keyword heading word] |
| --- | --- |
| S2 | (Prehypertension OR Pre-Hypertension OR Pre Hypertension).mp.[mp=title, abstract, heading word, drug trade name, original title, device manufacturer, device trade name, keyword heading word] |
| S3 | (Mindfulness OR mindfulness-based intervention* OR mindfulness-based stress reduction OR MBSR OR mindfulness-based cognitive therapy OR MBCT).mp.[mp=title, abstract, heading word, drug trade name, original title, device manufacturer, device trade name, keyword heading word] |
| S4 | (Randomized controlled trial OR randomi?ed OR RCT OR controlled clinical trial OR random allocation OR randomly).mp.[mp=title, abstract, heading word, drug trade name, original title, device manufacturer, device trade name, keyword heading word] |
| S5 | S1 OR S2 |
| S6 | S3 AND S4 AND S5 |

Search strategy for PsycINFO：

| #1 | MA Hypertension OR AB Blood Pressure, High OR AB Blood Pressures, High OR AB High Blood Pressure OR AB High Blood Pressures OR AB elevated blood pressure |
| --- | --- |
| #2 | MA Prehypertension OR AB Pre-Hypertension OR AB Pre Hypertension |
| #3 | MA Mindfulness OR AB mindfulness-based intervention* OR AB mindfulness-based stress reduction OR AB MBSR OR AB mindfulness-based cognitive therapy OR AB MBCT |
| #4 | PT Randomized controlled trial OR AB randomi?ed OR AB RCT OR AB controlled clinical trial OR AB random allocation OR AB randomly |
| #5 | #1 OR #2 |
| #6 | #3 AND #4 AND #5 |

For the China National Knowledge Infrastructure (CNKI), Wanfang databases, and VIP China Science, the following search strategy was used:

AB=高血压 AND AB=正念 OR 正念干预 OR 正念减压 OR 正念认知 OR MBSR OR MBCT AND AB=随机对照 OR随机 OR RCT

**Table S2. Basic characteristics of included studies.**

| First Author,  year | Country | Participants | | | | | Interve-ntion | Comp-arator | BP Assessment | | | Anxiety, Depression, Stress Outcomes | Main Findings |
| --- | --- | --- | --- | --- | --- | --- | --- | --- | --- | --- | --- | --- | --- |
|  |  | Study population | Total (IG:  CG) | Female （%） | Mean age  (SD) | Anti-  Hypertensive medication  (%) |  |  | Time  Points | Outcome | Devices |  |  |
| Palta,  2012 | USA | Hypertension | 20  (12:8) | 95 | IG: 72.3  (4.4) CG: 73.7  (5.8) | 90 | MBSR | AC:  social support group | Baseline,  Postinter-vention | Clinic SBP and DBP | Electronic blood pressure machine | Not reported | SBP:  IG exhibited 21.92mmHg lower than CG (p=0.020) DBP:  IG exhibited 16.70mmHg lower than CG (p=0.003) |
| Blom,  2013 | Canada | Prehypertensi-on or grade 1 hypertension | 101  (50:  51) | Total:62 IG: 64 CG: 63 | IG: 57  (12) WL: 55  (11) | Unmedicated | MBSR | WL | Baseline,  Week 12,  Week 24 | 24h ambulatory BP | Automated office BP measureme-nt device | Not reported | SBP:  IG -0.4 ± 6.7 mmHg  (P > 0.05)  DBP:  IG 0.04 ± 4.9 mmHg  (P > 0.05) |
| Hughes,  2013 | USA | Prehypertensi-on | 56  (28:  28) | Total:57 IG:61 CG:54 | 50.3 (6.5) | Unmedicated | MBSR | AC:  progressive muscle relaxation (PMR) training | Preinter-vention,  Postinter-vention | Clinic SBP/DBP  Ambulatory SBP and DBP | Automated oscillometric  BP device: Accutor Plus Oscillometric  BP Monitor | Not reported | SBP:  IG - 4.8mm Hg (p = .016) DBP:  IG - 1.9mm Hg (p = .008) MBSR did not result in larger decreases in ambulatory BP than in PMR. |
| Parswani,  2013 | India | Hypertension | 30  (15:  15) | all were males | IG: 47.27(12.15) CG: 50.60(8.21) | 100 | MBSR | TAU | Preinter-vention,  Postinter-vention,  Follow up at 3 months | Clinic SBP and DBP | Sphygmomanometer | Anxiety and depressive symptoms: HADS Perceived stress: PSS | SBP:  IG 135.67 ± 124.47 mmHg to 124.47 ± 8.97 (P < 0.001)  DBP:  IG 84.16 ± 5.48 mmHg to 81.60 ± 5.19 (P > 0.05)  Anxiety:  IG 7.87 ± 3.31 to 3.27 ± 1.27 (P < 0.001)  Depression:  IG 6.13 ± 2.03 to 3.33 ± 1.59 (P < 0.001)  Perceived stress:  IG 30.13 ± 4.87 to 19.60 ± 3.22 (P < 0.001 ) |
| Nejati, 2015 | Iran | Hypertension | 30  (15:  15) | Total: 46.67 IG:40 CG:53 | IG: 43.66 (5.14) CG: 43.13 (5.04) | 100 | MBSR | AC: yoga training | Preinter-vention,  Postinter-vention,  Follow up at 2 months | Clinic SBP and DBP | Not reported | Not reported | SBP:  IG 154.67 ± 7.54 to 138.11 ± 5.62 (p < 0.001)  DBP:  IG 90.58 ± 5.25 to 86.14 ± 5.65 (p <0.001) |
| Momeni,  2016 | Iran | Hypertension | 60  (30:  30) | 42 | Total: 47(7) IG: 49.16(6.31) CG:46.16  (6.27) | 100 | MBSR | WL | Preinter-vention,  Postinter-vention | Clinic SBP and DBP | Desk mercury sphygmomanometer | Perceived Stress: PSS | SBP  IG 134.16 ±7.99 mmHg to 118.33 ± 7.46 mmHg (P < 0.05)  DBP:  IG 81.66 ± 10.11 mmHg to 80.00 (P=0.61)  PS  IG 30.25 ± 9.59 to 16.74 ±4.76 (P< 0.05) |
| Kumar,  2017 | India | Grade 1 hypertension with Type 2 diabetes | 40  (20:  20) | Total：60.5 IG: 60.11 CG: 60 | 35-60  years | Total：68.4  IG: 66.67  CG: 70 | MBSR | WL | Preinter-vention,  Postinter-vention | Clinic SBP and DBP | Mercury sphygmomanometer | Not reported | SBP:  IG 144.56 ± 4.105 mmHg to 143.22 ± 3.828 mmHg (P = 0.006)  DBP:  IG 94.33 ± 3.447 mmHg to 93.67 ± 3.834 mmHg (P=0.187) |
| Márquez, 2018 | Spain | Prehypertension or grade 1 hypertension | 42  (24:  18) | 57.14 | 56.5  (7.77) | 65 | MBCT | AC: Health education | Preinter-vention,  week 4,  Posinter-  Vention,   Follow-up at week 20 | ClinicSBP and DBP  ABPM | OMRON M6 | Anxiety and depression: DASS-21  Perceived Stress: PSS-10 | Clinic SBP:  IG 130.54 ± 2.42 mmHg VS CG 133.21 ± 2.64 mmHg (P = 0.02)  Clinic DBP:  IG 84.25 ± 1.88 mmHg VS 87.11 ± 2.08 mmHg (P=0.89) |
| Liu,  2019 | China | Hypertension | 110  (57:  53) | Total: 43.6 IG: 43.9 CG：43.4 | IG: 56.19 (7.78) CG: 55.89 (7.77) | 100 | MBSR | TAU | Baseline,  Postinter-vention | Clinic SBP and DBP | Sphygmomanometer | Not reported | SBP:  IG 158.59 ±13.03 mmHg to 120.52 ± 14.89 mmHg (P < 0.01)  DBP:  IG 105.35 ± 9.62 mmHg to 80.77 ± 8.15 mmHg (P < 0.01) |
| An,  2021 | USA | Prehypertension or hypertension | 37  (20:  17) | Total:75 IG:70 CG:81 | IG: 58  (12.6) CG: 64  (9.0) | >60 | MAP | AC: Health Promo-tion Program | Baseline,  week 6,  Follow-up at week12 | Clinic SBP and DBP | BP monitor (Omron 3 series) | Not reported | SBP:  IG 138 ±15 mmHg to 119 ± 6 mmHg (P = 0.005) DBP:  IG 89 ±11 mmHg to 77 ± 7 mmHg (P = .003) |
| Yang,  2021 | China | Hypertension | 113  (57:  56) | Total: 49.56 IG: 50.88 CG: 48.21 | IG: 64.73 (2.03) CG: 64.56 (2.18) | 100 | MBSR | TAU | Preinter-vention,  Postinter-vention | Clinic SBP and DBP | Not reported | Anxiety: SAS Depression: SDS | SBP:  IG 161.48 ± 16.34 mmHg to 128.32 ± 12.06 mmHg (P<0.05)  DBP:  IG 102.17 ± 10.06 mmHg to 85.42 ± 8.13 mmHg (P<0.05)  Anxiety:  IG 60.11 ± 5.70 to 44.70 ± 4.31 (P<0.05) Depression:  IG 57.88 ± 5.53 to 42.06 ± 4.15 (P<0.05) |
| Babak，2022 | Iran | Hypertension | 80  (40:  40) | 100 | IG:  48.67  (1.42)  CG:  49.32  (2.31) | Not reported the proportion | MBSR | TAU | Preinter-vention,  Week 13 | Clinic SBP and DBP | Not reported | Anxiety: SAS Depression: DASS-21 | SBP:  IG -9.11 ± 0.85mmHg (P<0.001)  DBP:  IG -7.22 ± 0.67mmHg (P<0.001)  Anxiety:  IG -3.85 ± 0.15 (P<0.05)  Stress:  IG -4.76 ± 2.25 (P<0.05)  Depression:  IG -4.62 ± 2.11 (P<0.001) |

IG: intervention group；CG：control group；AC: active control；WL: wait list；TAU: treatment as usual；NS: non-significant；ABPM：ambulatory blood pressure monitoring；HADS: Hospital Anxiety and Depression Scale；PSS: Perceived Stress Scale；DASS-21：Depression, Anxiety and Stress Scales；SAS: Self-Rating Anxiety Scale；SDS：Self-rating depression scale

| **Table S3. Characteristics of interventions of included studies.** | | | | | | |
| --- | --- | --- | --- | --- | --- | --- |
| Study | Type of MBIs | Performer | Curriculum design | | | |
|  |  |  | Duration  of MBIs  (week) | Class length  (hour) | Home  Practice  (min) | Details of MBIs |
| Palta,  2012 | MBSR | interventionist trained in MBSR; through the Center for Mindfulness at the University of Massachusetts. | 8 | 1.5 | Yes, but the time was not reported. | The mindfulness-based program consisted of an opening meditation, sharing of successes, a review of home practices, a lesson to practice and apply mindfulness skills to self-care and interpersonal relationships, a guided breath meditation, a closing meditation, an invitation to do specific home practices, and a healthy fruit-and-vegetable snack. |
| Blom,  2013 | MBSR | 2 trained therapists | 8 | 2.5 | 45 | The MBSR program consisted of formal meditation, informal mindfulness practice, psycho-education activities, and self-monitoring/ reflection exercises. These therapeutic elements are explored through activities including but not limited to, gentle stretching and mindful yoga, a meditative body scan, mindful breathing, and mindful walking. |
| Hughes,  2013 | MBSR | MBSR therapist | 8 | 2.5 | Yes, but the time was not reported. | The MBSR program consisted of body scan exercise, sitting meditation, and yoga exercises. The daily homework exercises consisted of repeating body scan work, sitting meditation, and yoga exercises at home. Group members were provided audiotapes or CDs with guided MBSR exercises to assist their homework. |
| Parswani,  2013 | MBSR | NA | 8 | 1-1.5h | 30 | The MBSR program consisted of body scan meditation, sitting meditation, mindful walking, mindful eating, 3-min breathing space, mastery and pleasure activities and cognitive restructuring. Each participant was provided with an audio cassette with recorded instructions of mindfulness meditation and body scan meditation to practice 30 min of meditation at home. |
| Nejati, 2015 | MBSR | NA | 8 | NA | NA | The MBSR program consisted of sitting meditation, 3-min breathing exercise, conscious yoga, mindfulness of sounds and thoughts, etc. |
| Momeni,  2016 | MBSR | 1 licensed psychologist who was experienced in doing meditation exercises | 8 | 2.5 | 15-45 | The MBSR program consisted of a formal meditation and an educational component. Meditation techniques included mindful body scan, sitting meditation, walking meditation, and yoga. The participants were encouraged to use mindfulness exercises in their activities of daily living. |
| Kumar,  2017 | MBSR | NA | 8 | NA | NA | The MBSR program consisted of mindfulness breathing, body scan, sounds, and thoughts. |
| Márquez, 2018 | MBCT | 1 psychiatrist trained in MBCT | 8 | 2 | 45 | Group-based stress-reduction therapy based on mindfulness skills included mindfulness of breath, thoughts, bodily sensations, sounds and everyday activities. Patients were also encouraged to practice meditation at home for 45  min a day. |
| Liu,  2019 | MBSR | NA | 8 | NA | NA | The MBSR program consisted of mindful awareness, body scan, sitting meditation, 3-min breathing exercise, mindfulness of sounds and thoughts, unpleasant and pleasant experience etc. |
| An,  2021 | MAP | MARC-certified instructors | 6 | 2 | NA | The MAP program consisted of Introduction to mindfulness, listening, embodiment, and obstacles; working with pain; difficult emotions/positive emotions, thoughtful and mindful interactions; loving kindness. |
| Yang,  2021 | MBSR | Nurses | 8 | 0.75 | NA | The MBSR program consisted of introduction to mindfulness, sitting meditation, breathing exercise and body scan. |
| Babak,  2022 | MBSR | 1 psychologist skilled in MBSR | 8 | 2 | 45 | The MBSR program consisted of body scan exercises, sitting meditation, and yoga. Participants were advised to practice these skills at home for at least 45 minutes per day, 6 days per week. |

MBSR: mindfulness-based stress reduction; MBCT: mindfulness-based cognitive therapy; MAP: mindfulness awareness practice; MARC: Mindful (also known as mindfulness) Awareness Research Center; NA: not available.

**Appendices A**

Figure A-1: subgroup analysis of MBIs on SBP – by sample size.


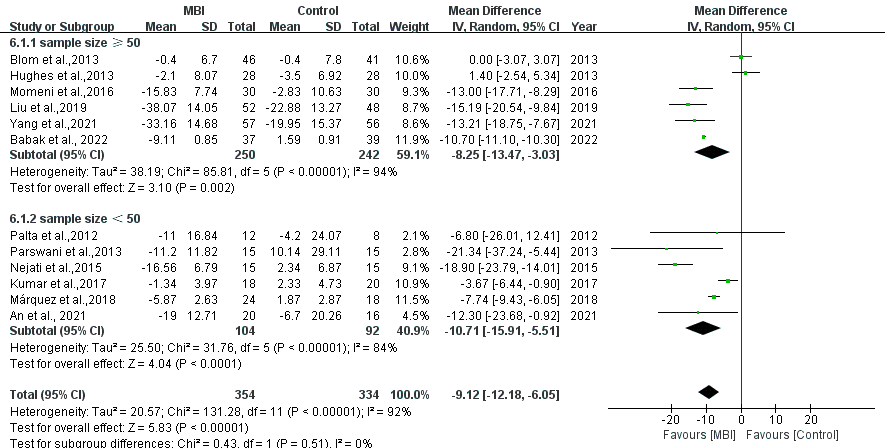


Figure A-2: subgroup analysis of mean difference of SBP – by control group.


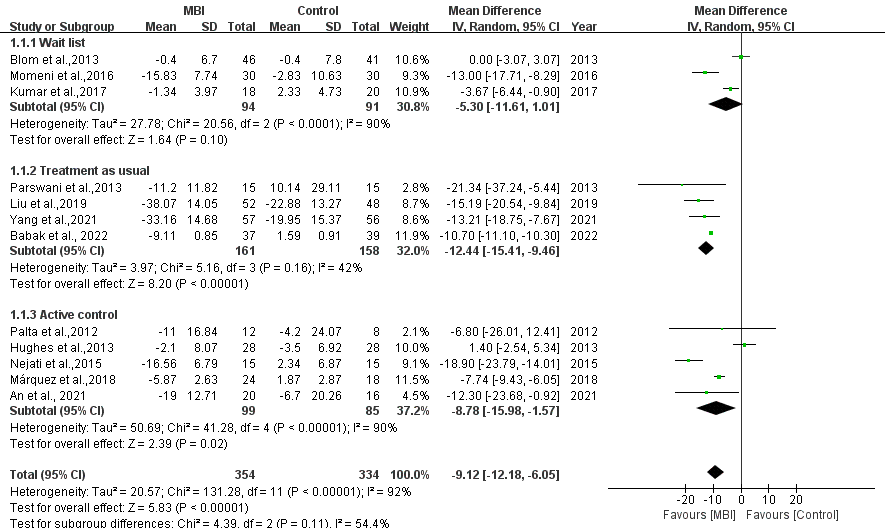


Figure A-3: subgroup analysis of mean difference of SBP – by baseline SBP.


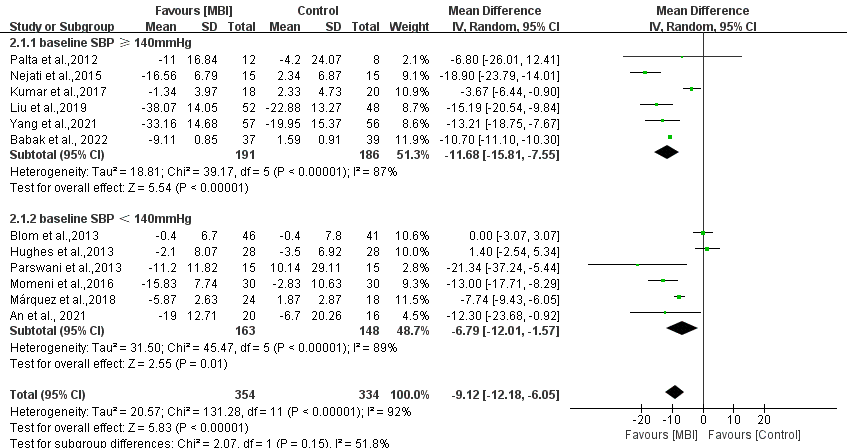


Figure A-4: subgroup analysis of mean difference of SBP – by female proportion.


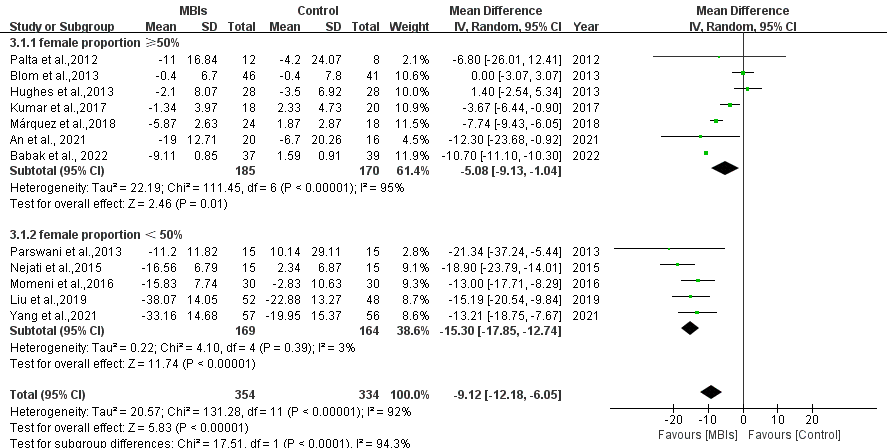


Figure A-5: subgroup analysis of mean difference of SBP – by use of antihypertensive drugs.


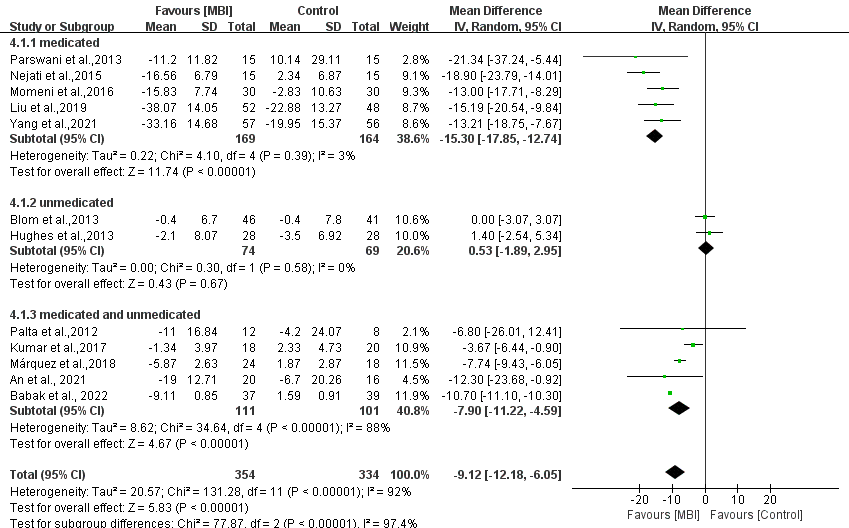


Figure A-6: subgroup analysis of mean difference of SBP – by the type of MBIs.


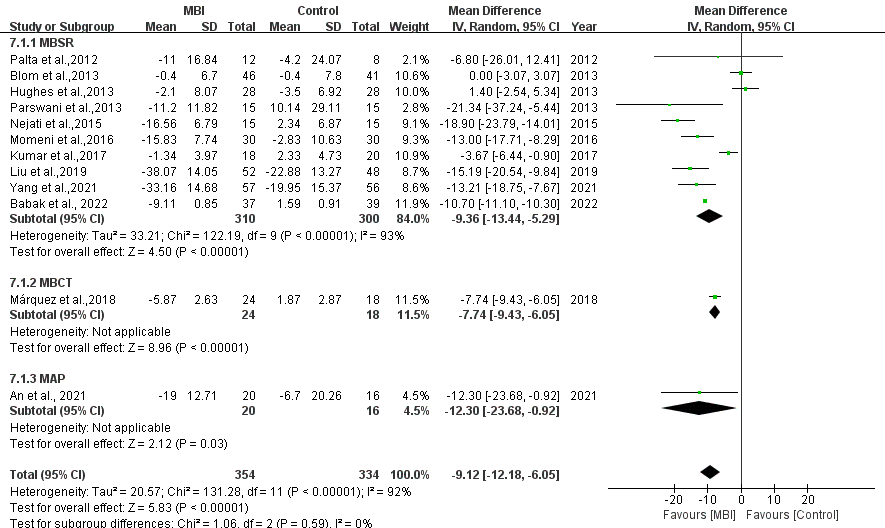


Figure A-7: subgroup analysis of mean difference of SBP – by the source of population.


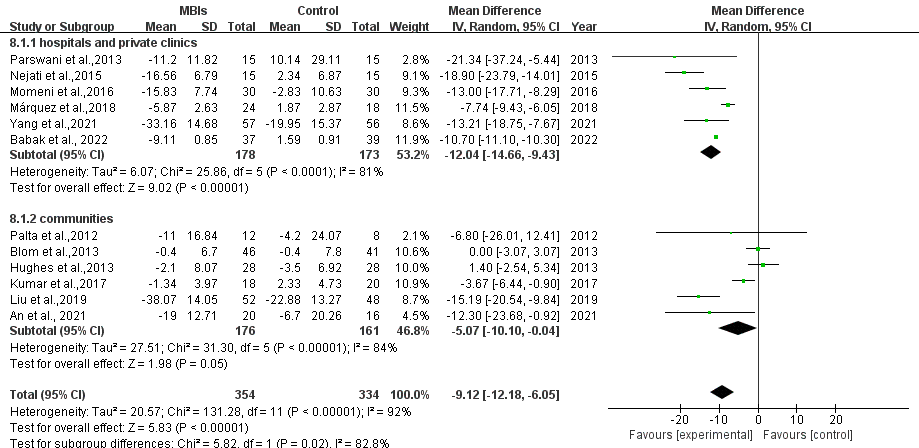


**Appendices B**

Figure B-1: subgroup analysis of MBIs on DBP – by sample size.


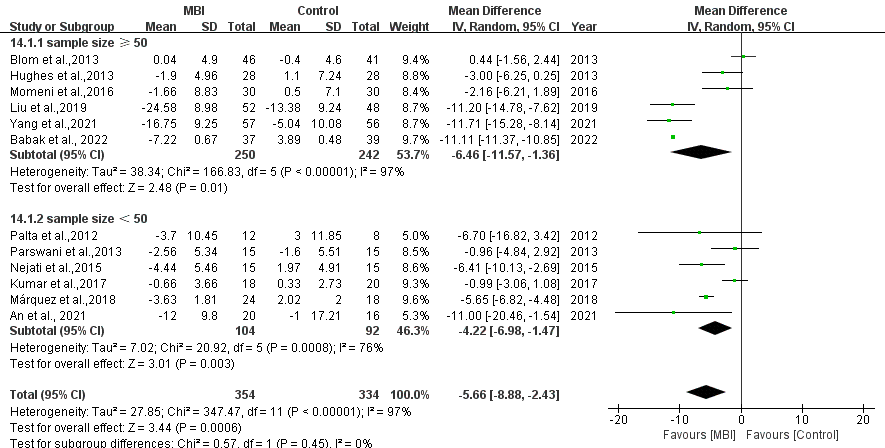


Figure B-2: subgroup analysis of mean difference of DBP – by control group.


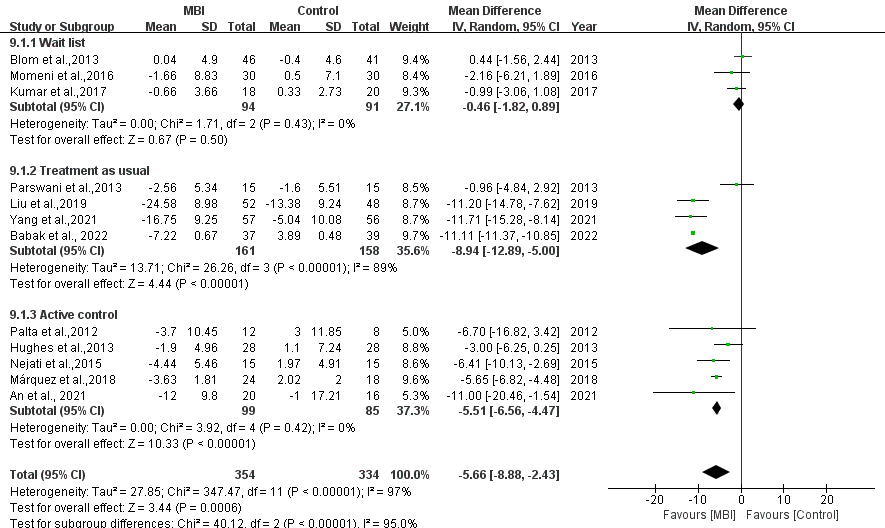


Figure B-3: subgroup analysis of mean difference of DBP – by baseline DBP.


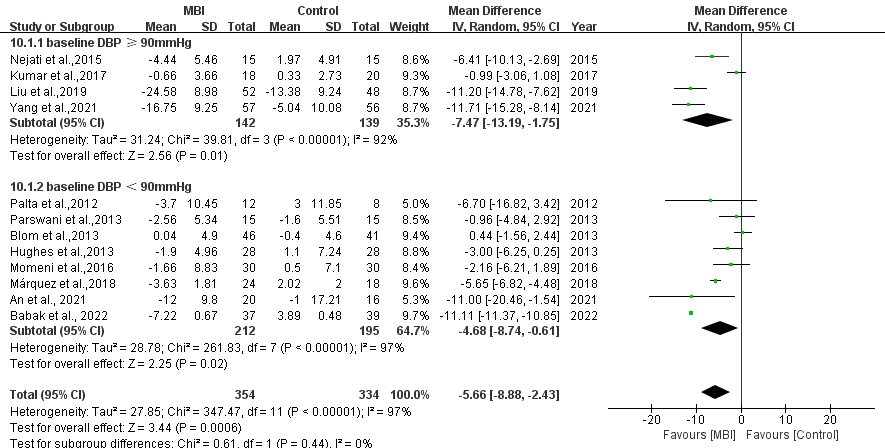


Figure B-4: subgroup analysis of mean difference of DBP – by female proportion.


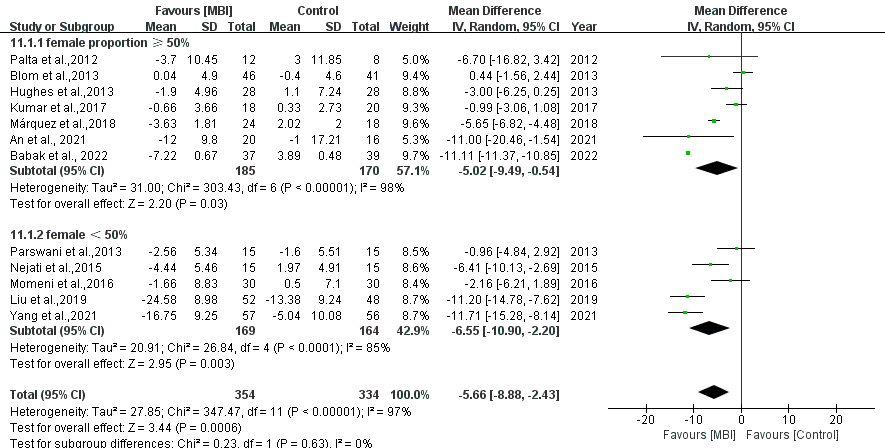


Figure B-5: subgroup analysis of mean difference of DBP – by use of antihypertensive drugs.


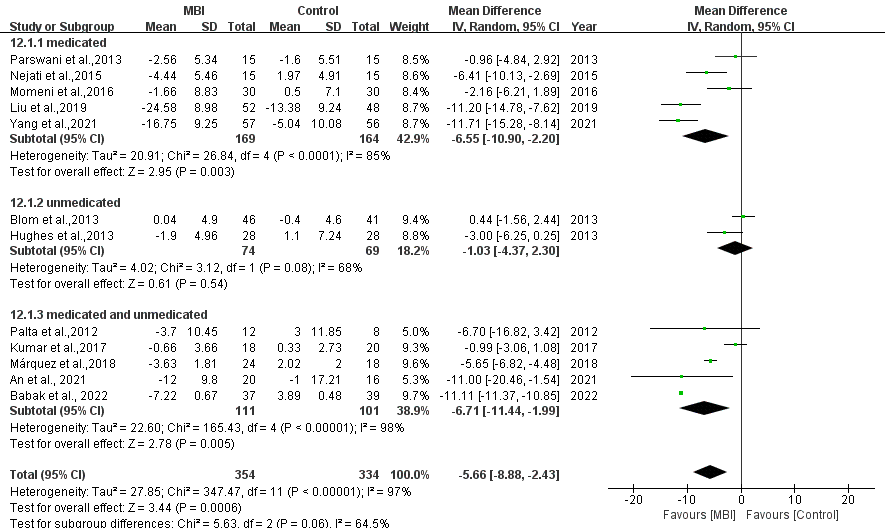


Figure B-6: subgroup analysis of mean difference of DBP – by the type of MBIs.


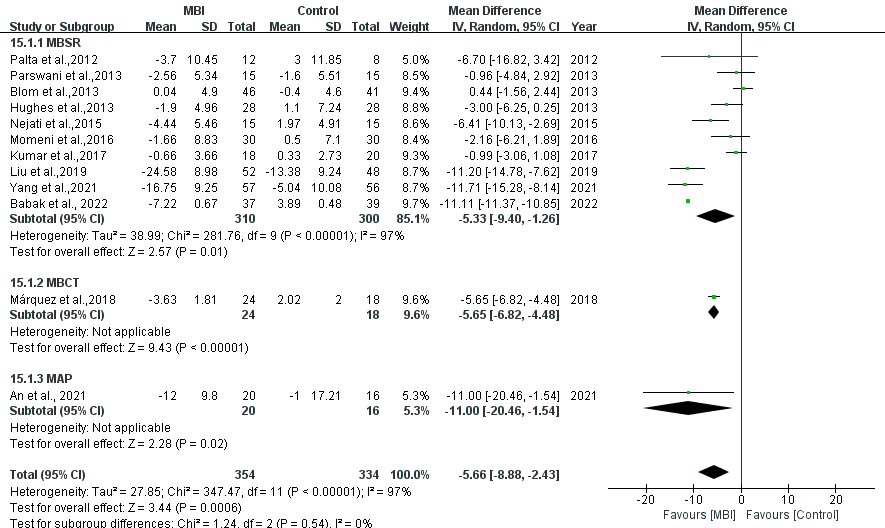


Figure B-7: subgroup analysis of mean difference of DBP – by the source of population.


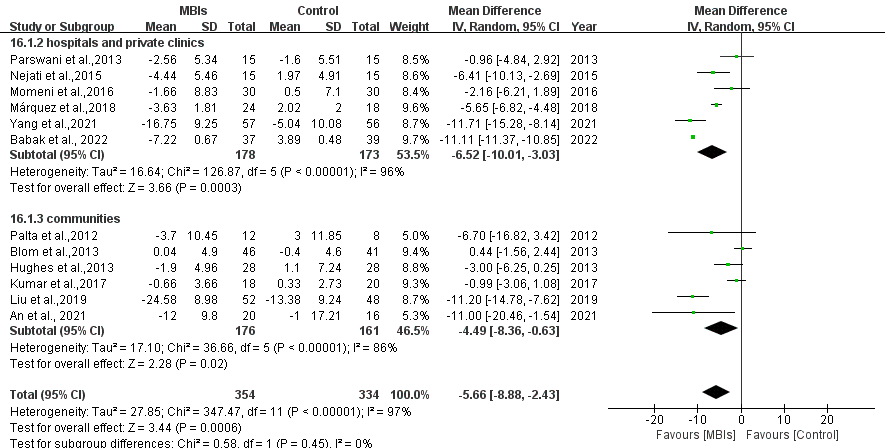

Supplement: Supplementary file 1 — Additional file 1. Supplementary Material. [file 12872_2024_3746_MOESM1_ESM.docx]
